# Increasing Cytosine Base Editing Scope and Efficiency With Engineered Cas9-PmCDA1 Fusions and the Modified sgRNA in Rice

**DOI:** 10.3389/fgene.2019.00379

**Published:** 2019-04-26

**Authors:** Ying Wu, Wen Xu, Feipeng Wang, Si Zhao, Feng Feng, Jinling Song, Chengwei Zhang, Jinxiao Yang

**Affiliations:** Beijing Key Laboratory of Maize DNA Fingerprinting and Molecular Breeding, Beijing Academy of Agriculture and Forestry Sciences, Beijing, China

**Keywords:** base editing, cytosine base editor, SpCas9, VQR, the modified sgRNA, rice

## Abstract

Base editors that do not require double-stranded DNA cleavage or homology-directed repair enable higher efficiency and cleaner substitution of targeted single nucleotides in genomic DNA than conventional approaches. However, their broad applications are limited within the editing window of several base pairs from the canonical NGG protospacer adjacent motif (PAM) sequence. In this study, we fused the D10A nickase of several *Streptococcus pyogenes* Cas9 (SpCas9) variants with *Petromyzon marinus* cytidine deaminase 1 (PmCDA1) and uracil DNA glycosylase inhibitor (UGI) and developed two new effective PmCDA1-based cytosine base editors (pBEs), SpCas9 nickase (SpCas9n)-pBE and VQR nickase (VQRn)-pBE, which expanded the scope of genome targeting for cytosine-to-thymine (C-to-T) substitutions in rice. Four of six and 12 of 18 target sites selected randomly in SpCas9n-pBE and VQRn-pBE, respectively were base edited with frequencies of 4–90% in T_0_ plants. The effective deaminase window typically spanned positions 1–7 within the protospacer and the single target C showed the maximum C-to-T frequency at or near position 3, counting the end distal to PAM as position 1. In addition, the modified single guide RNA (sgRNA) improved the base editing efficiencies of VQRn-pBE with 1.3- to 7.6-fold increases compared with the native sgRNA, and targets that could not be mutated using the native sgRNA were edited successfully using the modified sgRNA. These newly developed base editors can be used to realize C-to-T substitutions and may become powerful tools for both basic scientific research and crop breeding in rice.

## Introduction

Genome-wide association studies have shown that point mutations create elite trait variations in crop plants, and point mutagenesis is one of the main strategies for crop improvement (Henikoff and Comai, 2003; Zhao et al., 2011; Yin et al., 2017). The discovery and development of the CRISPR – Cas9 system (Doudna and Charpentier, 2014; Hsu et al., 2014; Shalem et al., 2015; Wang et al., 2016; Komor et al., 2017a) has provided a powerful genome engineering tool for generating point mutations in plants through precise irreversible base conversion (base editing) without the need for double-stranded DNA backbone cleavages or donor DNA templates (Komor et al., 2016; Nishida et al., 2016). Base editing is much cleaner and more efficient than current methods used in plants [e.g., targeting induced local lesions in genomes (TILLING) and conventional nuclease-mediated, homology-directed repair (HDR)-dependent genome editing] (Henikoff et al., 2004; Slade et al., 2005; Hess et al., 2017; Yang et al., 2017; Kim, 2018).

The first reported CBEs that mediate C-to-T conversion were developed in a wide variety of organisms by fusion of Cas9n with rat cytidine deaminase rAPOBEC1 or activation-induced cytidine deaminase ortholog PmCDA1 (Lu and Zhu, 2017; Li et al., 2017; Ren et al., 2017; Shimatani et al., 2017; Zong et al., 2017). Although highly efficient and useful, these CBEs were restricted to edit sites that contained NGG PAM sequences because of the common SpCas9n that was used (Anders et al., 2014; Nishimasu et al., 2014). This characteristic limited the base editing to a narrow window of several base pairs from the PAM distal region. To circumvent this limitation, several studies have reported new CBEs that use SpCas9 variants or Cas9 homologs that recognize expanded or altered PAMs to increase the targets suitable for base editing. In human cells, several engineered SpCas9 variants that accept NGA (VQR), NGCG (VRER), NGAG (EQR), or NG (xCas9 and SpCas9-NG) PAM sequences have been employed with rAPOBEC1 or activation-induced cytidine deaminase to generate new CBEs (Kim Y.B. et al., 2017; Hu et al., 2018; Nishimasu et al., 2018). In addition, the SaCas9, which recognizes the NNGRRT PAM, and its engineered variant SaKKH, which recognizes the NNNRRT PAM sequence, also have been used to create base editors that expand the editing capability of CBEs (Kim Y.B. et al., 2017).

Most of the Cas9s described above have been used to create new CBEs for plants; the exceptions are VRER and EQR (Hua et al., 2018; Qin et al., 2018; Endo et al., 2019). In addition, wild type SpCas9 was used to broaden the base editing targets for non-canonical NAG PAMs in plants (Hua et al., 2018). Among them, SpCas9n-NG, SaCas9n, and SaKKHn CBEs, both rAPOBEC1-based and activation-induced cytidine deaminase-based or PmCDA1-based, were successfully developed in rice (Qin et al., 2018; Endo et al., 2019). However, only rAPOBEC1-based CBEs were created with SpCas9n and VQRn, and only one editable target site was reported for each CBE (Hua et al., 2018). In this study, to better utilize SpCas9 and VQR to enlarge the base editing scope in rice, we developed two new effective PmCDA1-based CBEs (pBEs), SpCas9n-pBE, and VQRn-pBE. These two pBEs substantially broaden the target sites from those with NGG PAMs to those with NAG and NGA PAMs. Additionally, the editing efficiency of VQRn-pBE was further increased using the modified sgRNA.

## Materials and Methods

### Plasmid Construction

We modified the pCambia2300 plasmid to construct a vector called 2300-Spe. A schematic illustration of 2300-Spe vector construction is given in Supplementary Figure S1. Four fragments were digested at each end by restriction endonucleases to construct the SpCas9n-pBE-basic vector (Supplementary Figure S2). Then the four digested fragments together with the KpnI and SbfI digested 2300-Spe backbone were ligated using T4 ligase (NEB, Cat# M0202L) to generate SpCas9n-pBE-basic. Based on the SpCas9n-pBE-basic vector, specific point mutations described by Kleinstiver et al. (2015) were introduced into SpCas9n (D10A) using a Fast MultiSite Mutagenesis System (TransGen Biotech, Beijing, China) to generate VQRn-pBE-basic and VRERn-pBE-basic vectors. Target sequences were cloned before the sgRNA using BsaI according to Xie et al. (2015) to generate pBE constructions. The modified sgRNA linked with tRNA and the *Oryza sativa* U3 (OsU3) terminator was synthesized and digested with BamHI and HindIII, and used to replace the native sgRNA in the SpCas9n-pBE and VQRn-pBE constructions to obtain the corresponding pBEs with the modified sgRNA. Target sites in the same constructs are shown in Supplementary Table S1. The primers used in this study are listed in Supplementary Table S2.

### Rice Transformation

The wild type *Agrobacterium tumefaciens* strain LBA4404 (Weidi Biotech, Shanghai, China) was transformed by the resultant pBE constructs using a freeze/thaw method. Embryogenic calli induced from mature seeds of rice variety Nipponbare (*O. sativa* L. *japonica*. cv. Nipponbare) were used for the transformation, which was conducted as previously described (Hiei and Komari, 2008). After incubation with *Agrobacterium* for 10 min, the calli were recovered for 3 days and selected on 50 μg/ml hygromycin for 4 weeks to obtain resistant calli. Then, the resistant calli were transferred to regeneration medium (not containing hygromycin) to induce shoot regeneration for 1 month. When the shoots were 4–5 cm long, they were transferred to rooting medium for root induction for about 2 weeks to obtain T_0_ plants.

### DNA Extraction and Identification of Transgenic Resistant Calli and T_0_ Plants

Resistant calli and T_0_ plants were harvested for genomic DNA extraction using a DNA-quick Plant System kit (Tiangen Biotech, Beijing, China). The target locus was amplified by PCR with Cas9 specific primers (Supplementary Table S2) and samples with a 1150-bp nucleic acid band in agarose gel electrophoresis were identified as transgenic resistant calli or T_0_ plants.

### Mutant Identification

Several transgenic resistant calli and T_0_ plants in a single experiment were used to detect C-to-T conversions and indels. Target loci were amplified by specific primers and the PCR products were purified using an EasyPure PCR Purification Kit (TransGen Biotech). The PCR products were sent for Sanger sequencing (Tsingke Biological Technology, Beijing, China) to detect mutations. C-to-T frequency in calli or T_0_ plants was defined as the percentage of mutants with any target C-to-T substitution among all the transgenic samples. Indel frequency was defined as the percentage of mutants with any indels among the resulting C-to-T mutants. Single C-to-T frequency was defined as the percentage of mutants with C-to-T substitution at a specific single position among all the transgenic samples. Homozygous mutants were designated when all the mutations were homozygous. Frequency of mutant genotype was defined as the percentage of mutants with the same genotype among all the mutants.

### Detection of Off-Target Mutations

Five to eight single T_0_ plants, including base mutated lines and wild type lines, were selected for each off-target site detection. Potential off-target sites were searched on Cas-OFFinder (Bae et al., 2014) and amplified using the primers listed in Supplementary Table S2. The PCR products were purified using an EasyPure PCR Purification Kit (TransGen Biotech) and sent for Sanger sequencing (Tsingke Biological Technology) to detect off-target mutations.

## Results

### SpCas9n-pBE Enables Base Editing at NAG PAM Target Sites in Rice

**FIGURE 1:**
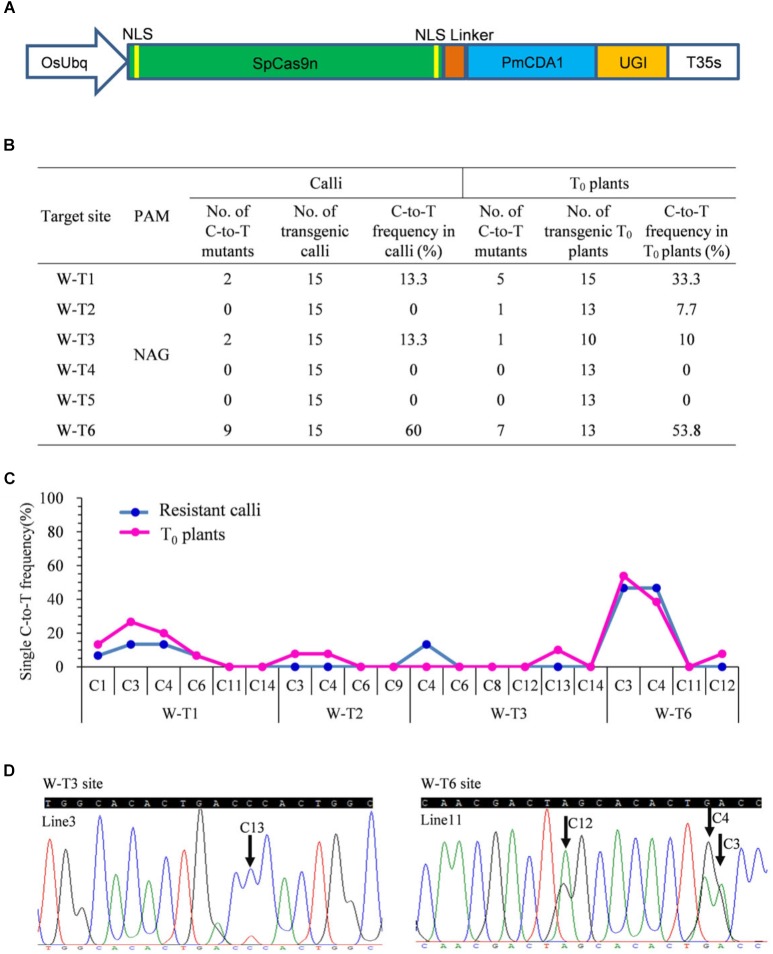
Base editing ability of SpCas9n-pBE in rice. **(A)** Schematic illustration of the SpCas9n-pBE construct. NSL, nuclear localization signal. **(B)** Frequencies of mutations induced by SpCas9n-pBE in resistant calli and T_0_ plants. **(C)** Frequencies of targeted single C-to-T substitutions in the targets edited by SpCas9n-pBE in resistant calli and T_0_ plants. **(D)** Sequencing chromatograms of T_0_ plants at the W-T3 and W-T6 sites of T_0_ transgenic lines 3 and 11, with the editing window extending positions 13 and 12 within the protospacer, respectively. Arrows indicate the edited bases. G-to-A conversions in the opposite strand are shown for the W-T6 target of line 11.

Previous studies revealed that the most widely used wild type SpCas9 enables efficient genome editing at target sites bearing both the canonical NGG PAM and the non-canonical NAG PAM in rice (Meng et al., 2018). Because the combination of PmCDA1 with SpCas9n leads to C-to-T substitutions at targets with the NGG PAM (Shimatani et al., 2017), we hypothesized that a SpCas9n base editor also could function at targets with the NAG PAM. We fused the D10A nickase of SpCas9 with PmCDA1 and UGI. The fusion protein was driven by the *O. sativa* ubiquitin (OsUbq) promoter and the corresponding cassette was introduced into our tRNA–sgRNA editing system to generate a pBE designated as SpCas9n-pBE (Figure 1A).

We first used a resistant rice calli system to determine the feasibility of C-to-T base editing using SpCas9n-pBE. Six targets with NAG PAMs from the *OsWaxy* gene, which encodes an enzyme essential in the biosynthesis of granule-bound starch, were selected (Supplementary Table S3). In three of the six targets, C-to-T base editing was detected with frequencies of 13.3–60% (Figure 1B). No indels were detected at any of the six on-target loci (Supplementary Table S3). Moreover, by analyzing the C-to-T frequency at each single C in the three edited targets, we found that the editing window spanned bases at positions 1 to 6 upstream of the PAM sequence (Figure 1C).

To further assess the use of SpCas9n-pBE in rice plants, the resistant calli were transferred to a regeneration culture to generate stable transgenic T_0_ plants. In T_0_ plants, four of the six target sites had C-to-T substitutions with frequencies of 7.7–53.8% (Figure 1B). Three of the target sites (W-T1, W-T3, and W-T6) were edited in both T_0_ plants and calli, whereas W-T2 was edited only in T_0_ plants with a frequency of 7.7% (Figure 1B). Among the four edited sites, indel was detected only at the W-T1 site in T_0_ plants (Supplementary Table S4). Except for the edited positions 13 and 12 in targets W-T3 and W-T6, respectively, the deamination window in T_0_ plants was consistent with that in the resistant calli (Figure 1C,D). Single and double-base substitutions were predominant in the edited targets in T_0_ plants. Triple or quadruple-base substitutions also were obtained for targets W-T6 and W-T1 (Supplementary Table S4). Furthermore, SpCas9n-pBE was able to be used for multiplex genome editing because two or three target sites were edited simultaneously in the same T_0_ plant line (Supplementary Table S5). Taken together, our results indicated that SpCas9n-pBE could broaden PAM recognition from NGG to NAG in rice.

### VQRn-pBE Enables Base Editing at NGA PAM Target Sites in Rice

**FIGURE 2:**
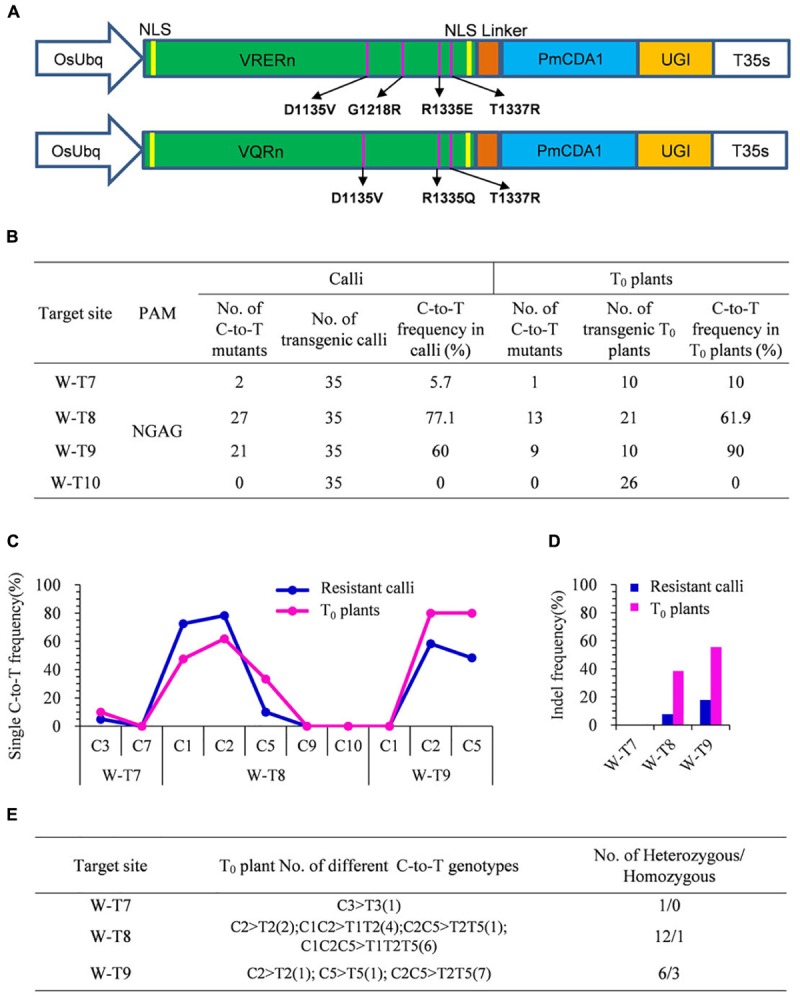
Base editing ability of VQRn-pBE at target sites on the *OsWaxy* gene in rice. **(A)** Schematic illustration of the VRERn-pBE (above) and VQRn-pBE (below) constructs. VQRn and VRERn were generated from SpCas9n with residue substitutions D1135V/R1335Q/T1337R and D1135V/G1218R/R1335E/T1337R, respectively. **(B)** Frequencies of mutations induced by VQRn-pBE at NGAG PAM target sites in resistant calli and T_0_ plants. **(C)** Frequencies of targeted single C-to-T substitutions in the targets edited by VQRn-pBE at NGAG PAM sites in resistant calli and T_0_ plants. **(D)** Indel frequencies in the targets edited by VQRn-pBE at NGAG PAM target sites in resistant calli and T_0_ plants. **(E)** Mutations induced by VQRn-pBE at NGAG PAM sites in T_0_ plants.

With different PAM specificities compared with SpCas9, VRER for NGCG PAMs, and VQR for NGA PAMs were reported to enable efficient genome editing of endogenous genes in zebrafish, human cells, and rice (Kleinstiver et al., 2015; Hu et al., 2016). To further enlarge the scope of C-to-T base editing in rice, we engineered two SpCas9 variants, VRER and VQR, then individually fused them with PmCDA1 and UGI to generate VRERn-pBE and VQRn-pBE (Figure 2A).

Eleven targets with NGCG PAMs were selected for VRERn-pBE editing, but the sequencing results showed none of them had C-to-T mutations in the resistant calli (Supplementary Table S6), implying VRERn-pBE had poor base editing activity in rice.

Because VQR mediated knockout mutations with a preference for NGAG > NGAT = NGAA in human cells (Kleinstiver et al., 2015), we tested the editing efficiency of VQRn-pBE for targets with NGAG PAMs. Four targets with NGAG PAMs from the *OsWaxy* gene were selected (Supplementary Table S7), and three of them were mutated successfully with frequencies of 5.7–77.1% in resistant calli and 10–90% in T_0_ plants (Figure 2B). The deamination windows spanned positions 1 to 5 of the protospacers, counting from the 5′ end of the target (Figure 2C). Indels were detected in two targets with relatively high substitution frequencies, and the corresponding indel frequencies were higher in T_0_ plants than in calli (Figure 2D). Single or double C conversions were the most common genotypes (Figure 2E). Among all the T_0_ base edited mutants, one homozygous mutant for W-T8 and three for W-T9 were obtained (Figure 2E). These results indicate VQRn-pBE could be used to enlarge the scope of base editing in rice.

We also detected the off-target effects of VQRn-pBE. Potential off-target sites that contained three to five mismatches with targets W-T7, W-T8, and W-T9 were chosen for the analysis (Supplementary Table S8). All these sites in T_0_ plants were sequenced and no mutations were detected among any of the selected off-target sites (data not shown).

**FIGURE 3:**
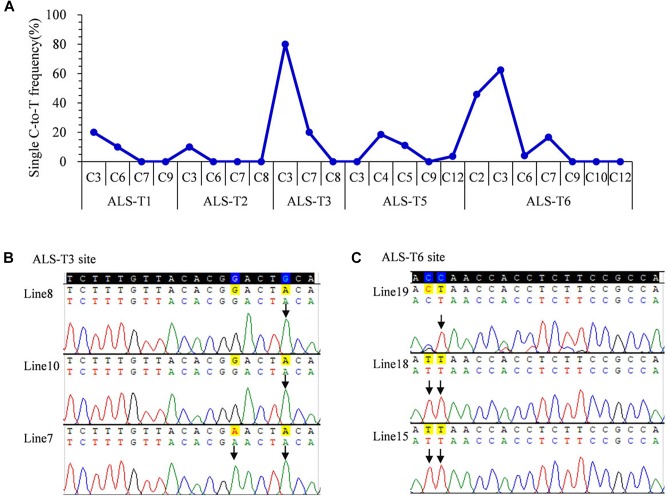
Base editing ability of VQRn-pBE at target sites on the *OsALS* gene in rice T_0_ plants. **(A)** Frequencies of targeted single C-to-T substitutions in the targets edited by VQRn-pBE at NGAG PAM target sites in T_0_ plants. **(B)** Sequencing chromatograms at the ALS-T3 target site of all three edited T_0_ lines with homozygous C-to-T conversions. The mutated bases are marked by arrows G-to-A conversions in the opposite strand are shown. **(C)** Sequencing chromatograms at the ALS-T6 target site of all three edited T_0_ lines with homozygous C-to-T conversions. The mutated bases are marked by arrows.

To avoid gene restriction and to confirm the capability of VQRn-pBE for base editing in rice, we used the *O. sativa* acetolactate synthase gene (*OsALS*), which encodes an essential enzyme in the biosynthesis of branched-chain amino acids. Six target sites with NGAG PAMs were selected (Supplementary Table S9). Among all the regenerated T_0_ events, five of the six sites were base edited with frequencies of 10–80%; the exception was the ALS-T4 site, which was not edited (Table 1). Indels were identified only in two targets with relatively high editing efficiencies (80 and 70.8%) (Table 1), similar to the results for the *OsWaxy* gene target sites. The effective deamination window typically spanned positions 2–7 within the protospacer, and the frequency of single C-to-T conversion was highest at or near position 3 (Figure 3A). Single C-to-T mutants were detected in all edited sites, and double C-to-T mutants were more than triple or quadruple mutants (Table 1). Additionally, three mutant lines contained homozygous substitutions were obtained in the ALS-T3 (C3 > T3 and C3C7 > T3T7) and ALS-T6 (C3 > T3 and C2C3 > T2T3) sites (Figure 3B,C and Table 1).

**Table 1:** Frequencies of mutations induced by VQRn-pBE at target sites on the *OsALS* gene in rice T_0_ plants.

Target site	No. of C-to-T mutants	No. of transgenic T_0_ plants	C-to-T frequency (%)	Indel frequency (%)	Genotype of C-to-T mutations	Heterozygous/homozygous
ALS-T1	2	10	20	0	C3 > T3(1); C3C6 > T3T6(1)	2/0
ALS-T2	1	10	10	0	C3 > T3(1)	1/0
ALS-T3	8	10	80	37.5	C3 > T3(6); C3C7 > T3T7(2)	5/3
ALS-T4	0	27	0	0		
ALS-T5	7	27	25.9	0	C4 > T4(3); C5 > T5(1); C12 > T12(1); C4C5 > T4T5(2)	7/0
ALS-T6	17	24	70.8	23.5	C2 > T2(1); C3 > T3(5); C7 > T7(1); C2C3 > T2T3(7); C2C3C7 > T2T3T7(2); C2C3C6C7 > T2T3T6T7(1)	14/3

Because VQRn-pBE mediated efficient base editing at sites that contained NGAG PAMs in rice, we determined whether it worked on targets with NGAT, NGAC, or NGAA PAMs. We selected 12 endogenous genomic target sites in the *OsWaxy* gene (Supplementary Table S10). We found that VQRn-pBE produced more C-to-T editing at target sites with the NGAT PAM than at target sites with the NGAC PAM in T_0_ plants, although the editing efficiencies were much lower in the former (Table 2). Similar to the results for the NGAG PAM target sites, VQRn-pBE produced indels only at the NGAC PAM W-T15 site, which had a relatively high base editing frequency (41.2% in T_0_ plants) (Supplementary Table S11). Single and double C-to-T mutants accounted for most of the detected mutants (Supplementary Table S11). VQRn-pBE produced no C-to-T editing at the four sites with the NGAA PAM in both calli and T_0_ plants (data not shown).

**Table 2:** Frequencies of mutations induced by VQRn-pBE at target sites on the *OsWaxy* gene with NGAT and NGAC PAMs in rice resistant calli and T_0_ plants.

Target site	PAM	Calli			T_0_ plants		
		No. of C-to-T mutants	No. of transgenic calli	C-to-T frequency in calli (%)	No. of C-to-T mutants	No. of transgenic T_0_ plants	C-to-T frequency in T_0_ plants (%)
W-T11	NGAT	2	15	13.3	1	25	4
W-T12		2	15	13.3	1	25	4
W-T13		0	15	0	1	17	5.9
W-T14		0	15	0	0	14	0
W-T15	NGAC	10	15	66.7	7	17	41.2
W-T16		0	15	0	0	17	0
W-T17		0	15	0	0	17	0
W-T18		0	15	0	0	16	0

### The Modified sgRNA Increases the Base Editing Efficiency of VQRn-pBE

**FIGURE 4:**
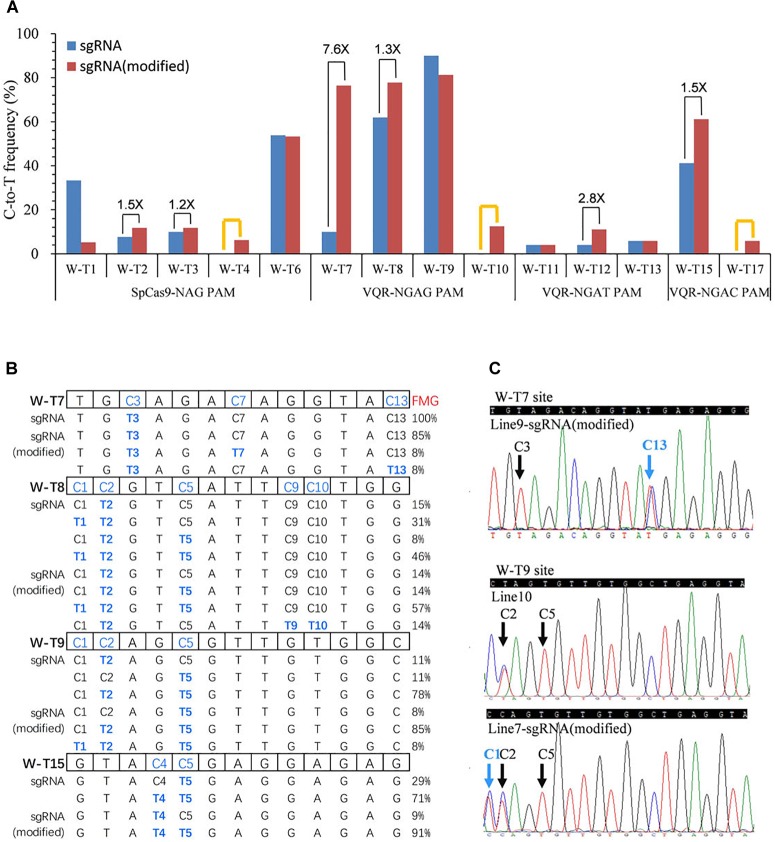
Base editing ability of SpCas9n-pBE and VQRn-pBE with the modified sgRNA in rice T_0_ plants. **(A)** C-to-T substitution efficiency of SpCas9n-pBE and VQRn-pBE at target sites with the native and modified sgRNAs. The PAM sequence of each target site is shown below the x axis. Black lines indicate targets where the editing frequency increased using the modified sgRNA compared with using the native sgRNA. Orange lines indicate targets that were not edited using the native sgRNA but were edited successfully using the modified sgRNA. **(B)** Comparison of mutant genotypes and frequency of mutant genotypes (FMGs) using VQRn-pBE with the native or modified sgRNA at four sites with the highest editing efficiencies. No mutations were detected in any cytidine residue located after position 13 in the targets, so the data from positions 14–20 upstream of the PAM sequence are omitted. **(C)** Sequencing chromatograms of T_0_ plants at the W-T7 target of line9 and W-T9 target of line 7, with additional C13 and C1 base substitutions obtained using the modified sgRNA. Blue arrows indicate the additional edited bases obtained using the modified sgRNA; black arrows indicate the edited bases obtained using the native sgRNA.

Several studies have reported that modified sgRNAs with a mutation in the streak of T and an extended duplex increased editing efficiency in mammalian cells and rice (Chen et al., 2013; Dang et al., 2015; Hu et al., 2017). To try to enhance the C-to-T substitution frequency, we modified the sgRNA as described previously in rice (Hu et al., 2017). We replaced the fourth T in the streak of T with C and extended the duplex by 5 bp (Supplementary Figure S3). Then, we used all 22 target sites in the *OsWaxy* gene with SpCas9n-pBE and VQRn-pBE with the modified sgRNA (Supplementary Tables S3, S7, S10). With SpCas9n-pBE, the modified sgRNAs showed equal or slightly higher editing frequencies (1.2 and 1.5 folds) than the native sgRNAs at the W-T2, W-T3, and W-T6 sites, efficiencies of 0–6.3% at the W-T4 site, and sharply decreased efficiencies (from 33.3% with the native sgRNA to 5.3% with the modified sgRNA) at the W-T1 site (Figure 4A). These results indicate that the modified sgRNA had an unsubstantial or small enhancement effect compared with native sgRNA for SpCas9n-pBE.

With VQRn-pBE, the modified sgRNA showed no base mutations at all four target sites with NGAA PAMs, which is similar to the results obtained with the native sgRNA (data not shown). However, base editing efficiencies were significantly enhanced by 1.3- to 7.6-fold for target sites with the other three PAMs using VQRn-pBE with the modified sgRNA (Figure 4A). Moreover, using the modified sgRNA produced C-to-T editing events with efficiencies of 0–12.5% and 5.9% in the W-T10 and W-T17 sites, but equal or slightly decreased efficiencies in the W-T9, W-T11, and W-T13 sites compared with using the native sgRNA (Figure 4A). These results indicate that the modified sgRNA was more effective in promoting the base editing efficiency of VQRn-pBE than that of SpCas9n-pBE.

Interestingly, except for the four targets with NGAG PAMs, no indels were identified in any of the targets with the other three PAMs using the modified sgRNA (Supplementary Figure S4). We compared the mutant genotypes of four sites with the highest editing efficiencies using VQRn-pBE with the native or modified sgRNAs. We found that the frequencies of single C-to-T mutations decreased and the frequencies of double or multiple C-to-T mutations increased when the modified sgRNA was used (Figure 4B). New genotypes also were produced in some target sites, such as C3C13 > T3T13 at the W-T7 site and C1C2C5 > T1T2T5 at the W-T9 site (Figure 4B,C). Moreover, the editing window was enlarged to position 13 and 10 within the protospacer at W-T7 and W-T8 sites when the modified sgRNA was used (Figure 4B,C).

Because the base editing efficiency of VQRn-pBE was increased with the modified sgRNA, we also detected its off-target effects. The potential off-target sites were the same as those analyzed using the native sgRNA (Supplementary Table S8). The sequences were amplified in T_0_ plants for Sanger sequencing and no mutations were found at any of the off-target loci tested (data not shown).

## Discussion

Cytosine base editors are powerful new tools for targeted base editing in cells and organisms (Hess et al., 2017; Kim, 2018). The NGG PAM requirement of canonical SpCas9 greatly limits the targeting scope of CBEs. In this study, by fusing SpCas9n and its variants VRERn and VQRn with PmCDA1, we obtained two effective base editors, SpCas9n-pBE, and VQRn-pBE. Consistent results from both calli and T_0_ plants confirmed the editing ability of both these base editors. About 66.7% of the selected target sites were base edited using SpCas9n-pBE (4/6) and VQRn-pBE (12/18) with frequencies of 4–90% in T_0_ plants (Figure 1B, 2B and Tables 1, 2). Therefore, the pBEs enlarged the range of cytidine base editing from NGG PAM to NAG and NGA PAMs in rice.

Although VRERn fused with rAPOBEC1 was active in human cells (Kim Y.B. et al., 2017), no mutations were detected among the selected targets in rice with VRERn-pBE. This may be explained by the different genome environments in plant and human cells and the different deaminase base editing systems used. This result suggests that not all Cas9 variants that perform well in human cells will perform well in rice, and other Cas9 variants need to be tested before they are applied in plant.

In human cells, VQR produced different cleaving efficiency at sites that contained NGAN PAMs as follows: NGAG > NGAT = NGAA > NGAC (Kleinstiver et al., 2015). Therefore, we designed four NGAN PAMs for rice and tested the base editing activity of VQRn-pBE for the different target sites. Mutations were detected in 80% (8/10) of the target sites with the NGAG PAM, and in 75% (3/4) and 25% (1/4) at the target sites with the NGAT and NGAC PAMs. No mutations were found in target sites with the NGAA PAM. These results imply that VQRn-pBE may have a strong preference for targets with NGAN PAMs as follows: NGAG > NGAT > NGAC > NGAA. However, the editing efficiencies were very low (4–7.1%) in the 75% edited targets with a NGAT PAM and much higher (41.2%) in the 25% edited targets with a NGAC PAM. Hence, we can not conclude the editing efficiency of VQRn-pBE was higher for targets with the NGAT PAM than for targets with the NGAC PAM. Recently, Hua and coworkers fused VQRn with rAPOBEC1 to achieve C-to-T substitutions in rice and reported 71.4% editing efficiency at one target site with a NGAG PAM (Hua et al., 2018). This is similar with our results with VQRn-pBE, which was more efficient at sites harboring NGAG PAMs.

For both SpCas9n-pBE and VQRn-pBE, the editing window typically spanned positions 1 to 7 within the protospacer, and the target C at or near position 3 showed the highest C-to-T substitution frequency. This result is a little different from the reported Cas9n-plant base editor composed of rAPOBEC1, Cas9n, and UGI in rice, in which the deamination window spanned positions 3–9, and the target C at or near position 7 showed the highest editing efficiency (Zong et al., 2017). Moreover, indels seem to be produced at the targets with relatively high editing efficiencies. Introduction of the Gam protein of bacteriophage Mu (Komor et al., 2017b) can be tested to reduce the indel frequency and improve product purity in the future.

Modified sgRNAs were reported to improve knock out activity in mammalian cells and in rice when combined with wild type SpCas9 or its VQR variant (Chen et al., 2013; Hu et al., 2017). In our study, targets that were not mutated with the native sgRNA were successfully base edited with the modified sgRNA. Moreover, the enhanced efficiency using the modified sgRNA was more predominant for VQRn-pBE than for SpCas9n-pBE. Analysis of the mutant genotypes in targets with high editing frequencies revealed changes that occurred when the modified sgRNA was used: (i) the frequencies of single C-to-T conversions decreased and double or multiple C-to-T substitutions increased, and (ii) mutants with novel single C-to-T conversions in different positions or with new multiple C-to-T mutations at various positions were obtained. Together, these results suggest that the modified sgRNA could be used to increase the editing frequency of VQRn-pBE in rice under applicable circumstances.

Other natural or evolved CRISPR nucleases with different PAM requirements that can broaden the editable targets include *Lachnospiraceae bacterium* Cpf1, *Neisseria meningitidis* Cas9, *Campylobacter jejuni* Cas9, and *Streptococcus thermophilus* Cas9 (Hou et al., 2013; Glemzaite et al., 2015; Kim E. et al., 2017; Tang et al., 2017; Li et al., 2018; Zhong et al., 2018). Recently, a human cytidine deaminase APOBEC3A was used to generate an effective base editor in mammalian cells and plants (Gehrke et al., 2018; Zong et al., 2018). To further expand the scope of base editing in rice, all the above CRISPR nucleases and human APOBEC3A should be tested in the future.

## Conclusion

In this study, we described two efficient PmCDA1-based CBE systems, SpCas9n-pBE and VQRn-pBE, that will help to expand the scope of cytosine base editing in rice. The effective deamination window typically spanned positions 1–7 of the protospacer and the target single C showed the highest editing frequency at or near position 3. The mutant genotypes were mainly single or double C-to-T substitutions. Furthermore, the editing efficiency of VQRn-pBE was increased by the modified sgRNA. These base editors will be useful tools for scientific research and crop breeding in rice.

## Author Contributions

JY, CZ, and YW designed the experiments and wrote the manuscript. YW, FW, SZ, FF, and JS performed all the experiments. WX and FW analyzed the results. JY supervised the project. All authors read and approved the final manuscript.

## Conflict of Interest Statement

The authors submitted a patent application based on the results reported in this paper.

## Abbreviations

Cas9: CRISPR-associated protein 9
CBE: cytosine base editor
CRISPR: clustered regularly interspaced short palindromic repeats
C-to-T: cytosine-to-thymine
EQR: engineered SpCas9 variant with residue substitutions D1135E/R1335Q/T1337R
Indel: insertion and deletion
PAM: protospacer adjacent motif
pBE: PmCDA1-based cytosine base editor
PmCDA1: *Petromyzon marinus* cytidine deaminase1
rAPOBEC1: rat cytidine deaminase APOBEC1
SaCas9: *Staphylococcus aureus* Cas9
SaCas9n: SaCas9 nickase
SaKKH: engineered SaCas9 variant with residue substitutions E782K/N968K/R105H
SaKKHn: SaKKH nickase
sgRNA: single guide RNA
SpCas9: *Streptococcus pyogenes* Cas9
SpCas9n: SpCas9 nickase
SpCas9-NG: engineered SpCas9 variant with residue substitutions R1335V/L1111R/D1135V/G1218R/E1219F/A1322R/T1337R
SpCas9n-NG: SpCas9-NG nickase
tRNA: transfer RNA
UGI: uracil DNA glycosylase inhibitor
VQR: engineered SpCas9 variant with residue substitutions D1135V/R1335Q/T1337R
VQRn: VQR nickase
VRER: engineered SpCas9 variant with residue substitutions D1135V/G1218R/R1335E/T1337R
VRERn: VRER nickase
xCas9: engineered SpCas9 variant with residue substitutions E480K/E543D/E1219V.
