# Supplementary material for: Increasing Cytosine Base Editing Scope and Efficiency With Engineered Cas9-PmCDA1 Fusions and the Modified sgRNA in Rice
Source: Front Genet. 2019 Apr 26;10:379. doi: 10.3389/fgene.2019.00379 (PMC6512751; doi:10.3389/fgene.2019.00379)
Supplement: Supplementary file 1 [file Data_Sheet_1.docx]

Supplementary Material

Increasing cytosine base editing scope and efficiency with engineered Cas9-PmCDA1 fusions and the modified sgRNA in rice

Ying Wu, Wen Xu, Feipeng Wang, Si Zhao, Feng Feng, Jinling Song, Chengwei Zhang* and Jinxiao Yang*

*** Correspondence:**

Dr. Chengwei Zhang

zhangchengwei2017@126.com

Dr. Jinxiao Yang

yangjinxiao@maizedna.org Corresponding Author: email@uni.edu

# Supplementary Data

**a**

**SpCas9n(D10A) without TAG**

atggactacaaggaccacgacggggattacaaagaccacgacatagactacaaggatgacgatgacaaaatggcaccgaagaaaaaaaggaaggtcggaatccatggcgttccagctgccgataagaaatattccatcggactcgccattggcacgaatagcgtcggatgggctgttattactgatgagtacaaagttccgtctaagaagttcaaggtgctgggcaacacagaccgccacagcataaagaaaaatctcatcggtgcactccttttcgatagtggggagactgcagaagcgacaagattgaaaaggactgcgagaaggcgctatacacggcgtaagaatagaatctgctaccttcaggagattttctctaacgaaatggctaaggtcgatgacagtttctttcatagacttgaggaatcgttcttggttgaggaggataagaaacatgagaggcacccgatatttggaaacatcgtggatgaggtcgcatatcatgaaaagtaccccacaatctaccacctgagaaagaaactcgttgattccaccgacaaagcggatttgagactcatctacctcgctcttgcccatatgataaagttccgcggacactttctgatcgagggcgacctcaaccctgataatagcgacgtcgataagctcttcatccagttggttcaaacctacaatcagctctttgaggaaaacccaattaatgctagtggagtggatgcaaaagcgatactgtcggccagactctccaagagcagaaggttggagaacctgatcgctcaacttcctggagaaaagaaaaacggtctttttgggaatttgattgccttgtctctgggcctcacaccaaacttcaagtcaaattttgacctcgctgaggatgccaaacttcagttgtctaaggatacctatgatgacgatcttgacaatttgctggcacaaattggcgaccagtacgcggatctgttcctcgcagcgaagaatctgagtgatgctattctcctttcggacatactcagggttaacactgagatcacaaaagcacctttgagtgcgtcgatgattaagcgctatgatgaacatcaccaagacctcactttgctgaaggcccttgtgcggcagcaattgccagagaagtacaaagaaatcttctttgaccaatctaagaacggatacgctggctatattgatggaggagcttctcaggaggaattctataagtttatcaaacctatacttgagaagatggatggtacagaggaactccttgttaaattgaacagagaagatttgctgcgcaagcaacggacctttgacaacggatcaattccgcatcagatacacctcggcgagcttcatgccatccttcgccggcaggaagatttctacccctttttgaaggacaaccgcgagaagatagaaaaaatccttacgttccggattccttactatgtgggtccattggcaagggggaattcccgctttgcgtggatgactcggaaaagcgaggaaactatcacaccgtggaacttcgaggaagttgtggacaagggagcttctgcccaatcattcattgagaggatgactaacttcgataagaacctgccgaacgagaaagttctccccaagcactccctcctttacgagtatttcaccgtgtataacgaacttacgaaggttaaatacgtgactgagggtatgaggaagccagcattcttgagcggggaacaaaagaaagcgattgttgatttgctgtttaaaactaatcgcaaggtgacagtcaagcagctcaaagaggattatttcaagaaaattgaatgtttcgactctgtggagatatcaggagtcgaagataggtttaacgcttcccttggcacataccatgacctccttaagatcattaaggacaaagatttcctggataacgaggaaaatgaggacatcctcgaagatattgttcttaccttgacgctgtttgaggatcgcgaaatgatcgaggaacggcttaagacgtatgctcacttgttcgacgataaggttatgaagcagctcaagcgtagaaggtacactggatggggccgtctgtctagaaagctcatcaacggaatacgtgataaacaaagtggcaagacaattttggattttctgaagtcggacggattcgccaacagaaattttatgcagctgattcatgacgatagtctcaccttcaaagaggacatacagaaggctcaagtgagtggtcaaggggattcgctgcatgaacacatcgcaaacctcgcgggttcaccggccataaagaaaggaatccttcaaactgttaaggtcgttgatgagttggttaaagtgatgggtaggcacaagcccgaaaacatagtgatcgagatggctcgcgaaaatcagactacacaaaaagggcagaagaactctcgcgagcggatgaaaaggattgaggaaggaatcaaggaactgggctcacagattctcaaagagcatccagtcgaaaacacacagctgcaaaatgagaagctctatctttactatctccaaaatggccgggacatgtatgttgatcaggagcttgacatcaaccgtttgtccgactatgatgtggaccacattgtcccgcaatctttccttaaggacgattcaatcgataataaggtgttgacccggagcgataaaaaccgtggaaagtctgacaatgtcccttcagaggaagtggttaagaagatgaagaactactggagacaattgctgaatgcaaaactgatcacacagagaaagttcgacaacctcaccaaagcagagagaggtgggctcagtgaacttgataaagcgggcttcattaagcgtcagctcgttgagactagacagatcacgaagcatgtcgcgcagattttggattcgcggatgaacacgaagtacgacgagaatgataaactgatacgtgaagtcaaggttatcactcttaagtccaaattggtgagcgatttcagaaaggacttccaattctataaggtcagggagatcaacaattatcatcacgctcacgatgcctaccttaatgctgttgtggggaccgcccttattaagaaataccctaaattggagtctgaattcgtttacggggattataaggtctacgacgttaggaaaatgatagctaagagtgagcaggagatcggtaaagcaactgcgaagtatttcttttactcgaacatcatgaatttctttaagaccgagataacgctggcaaatggcgaaattagaaagaggcctctcatagagactaacggtgagacaggggaaatcgtctgggataagggtagggactttgcgacagtgcgcaaggtcctctctatgccgcaagttaatattgtgaagaaaaccgaggtgcagacgggaggcttctccaaggaaagcatacttcccaaacggaactctgataagttgatcgctcgtaagaaagattgggaccctaagaaatatggtgggttcgattccccaactgttgcttacagcgtgctggtcgttgccaaggtcgagaagggtaaatccaagaaactcaaaagcgttaaggaactccttgggattactatcatggagagatcttcattcgaaaagaatcctatcgactttcttgaggccaaaggatataaggaagttaagaaagatctgataatcaaactcccaaagtactcattgtttgagctggaaaacggcaggaagcgcatgcttgcttccgccggagagttgcagaaagggaacgagttggctctgccttctaagtatgttaacttcctctatcttgcctctcattacgagaagctcaaaggctcaccagaggacaacgaacagaaacaactttttgtcgagcaacataagcactatttggatgagattatagaacagatcagtgaattctcgaaaagggttatccttgcagatgcgaatcttgacaaggtgttgtctgcatacaacaaacatagagataagccgatcagggagcaagcggaaaatatcattcacctcttcactcttacaaacttgggtgctcccgctgccttcaagtattttgataccacgattgaccggaaacgttacacctcaacgaaggaggtgctggatgccaccctcatccaccaatctattaccggactctacgagactagaatcgatctctcacagctcggcggggataaaagaccagcagcgacgaaaaaggcaggacaggctaagaagaagaaa

**b**

**VQRn(D10A) without TAG**

atggactacaaggaccacgacggggattacaaagaccacgacatagactacaaggatgacgatgacaaaatggcaccgaagaaaaaaaggaaggtcggaatccatggcgttccagctgccgataagaaatattccatcggactcgccattggcacgaatagcgtcggatgggctgttattactgatgagtacaaagttccgtctaagaagttcaaggtgctgggcaacacagaccgccacagcataaagaaaaatctcatcggtgcactccttttcgatagtggggagactgcagaagcgacaagattgaaaaggactgcgagaaggcgctatacacggcgtaagaatagaatctgctaccttcaggagattttctctaacgaaatggctaaggtcgatgacagtttctttcatagacttgaggaatcgttcttggttgaggaggataagaaacatgagaggcacccgatatttggaaacatcgtggatgaggtcgcatatcatgaaaagtaccccacaatctaccacctgagaaagaaactcgttgattccaccgacaaagcggatttgagactcatctacctcgctcttgcccatatgataaagttccgcggacactttctgatcgagggcgacctcaaccctgataatagcgacgtcgataagctcttcatccagttggttcaaacctacaatcagctctttgaggaaaacccaattaatgctagtggagtggatgcaaaagcgatactgtcggccagactctccaagagcagaaggttggagaacctgatcgctcaacttcctggagaaaagaaaaacggtctttttgggaatttgattgccttgtctctgggcctcacaccaaacttcaagtcaaattttgacctcgctgaggatgccaaacttcagttgtctaaggatacctatgatgacgatcttgacaatttgctggcacaaattggcgaccagtacgcggatctgttcctcgcagcgaagaatctgagtgatgctattctcctttcggacatactcagggttaacactgagatcacaaaagcacctttgagtgcgtcgatgattaagcgctatgatgaacatcaccaagacctcactttgctgaaggcccttgtgcggcagcaattgccagagaagtacaaagaaatcttctttgaccaatctaagaacggatacgctggctatattgatggaggagcttctcaggaggaattctataagtttatcaaacctatacttgagaagatggatggtacagaggaactccttgttaaattgaacagagaagatttgctgcgcaagcaacggacctttgacaacggatcaattccgcatcagatacacctcggcgagcttcatgccatccttcgccggcaggaagatttctacccctttttgaaggacaaccgcgagaagatagaaaaaatccttacgttccggattccttactatgtgggtccattggcaagggggaattcccgctttgcgtggatgactcggaaaagcgaggaaactatcacaccgtggaacttcgaggaagttgtggacaagggagcttctgcccaatcattcattgagaggatgactaacttcgataagaacctgccgaacgagaaagttctccccaagcactccctcctttacgagtatttcaccgtgtataacgaacttacgaaggttaaatacgtgactgagggtatgaggaagccagcattcttgagcggggaacaaaagaaagcgattgttgatttgctgtttaaaactaatcgcaaggtgacagtcaagcagctcaaagaggattatttcaagaaaattgaatgtttcgactctgtggagatatcaggagtcgaagataggtttaacgcttcccttggcacataccatgacctccttaagatcattaaggacaaagatttcctggataacgaggaaaatgaggacatcctcgaagatattgttcttaccttgacgctgtttgaggatcgcgaaatgatcgaggaacggcttaagacgtatgctcacttgttcgacgataaggttatgaagcagctcaagcgtagaaggtacactggatggggccgtctgtctagaaagctcatcaacggaatacgtgataaacaaagtggcaagacaattttggattttctgaagtcggacggattcgccaacagaaattttatgcagctgattcatgacgatagtctcaccttcaaagaggacatacagaaggctcaagtgagtggtcaaggggattcgctgcatgaacacatcgcaaacctcgcgggttcaccggccataaagaaaggaatccttcaaactgttaaggtcgttgatgagttggttaaagtgatgggtaggcacaagcccgaaaacatagtgatcgagatggctcgcgaaaatcagactacacaaaaagggcagaagaactctcgcgagcggatgaaaaggattgaggaaggaatcaaggaactgggctcacagattctcaaagagcatccagtcgaaaacacacagctgcaaaatgagaagctctatctttactatctccaaaatggccgggacatgtatgttgatcaggagcttgacatcaaccgtttgtccgactatgatgtggaccacattgtcccgcaatctttccttaaggacgattcaatcgataataaggtgttgacccggagcgataaaaaccgtggaaagtctgacaatgtcccttcagaggaagtggttaagaagatgaagaactactggagacaattgctgaatgcaaaactgatcacacagagaaagttcgacaacctcaccaaagcagagagaggtgggctcagtgaacttgataaagcgggcttcattaagcgtcagctcgttgagactagacagatcacgaagcatgtcgcgcagattttggattcgcggatgaacacgaagtacgacgagaatgataaactgatacgtgaagtcaaggttatcactcttaagtccaaattggtgagcgatttcagaaaggacttccaattctataaggtcagggagatcaacaattatcatcacgctcacgatgcctaccttaatgctgttgtggggaccgcccttattaagaaataccctaaattggagtctgaattcgtttacggggattataaggtctacgacgttaggaaaatgatagctaagagtgagcaggagatcggtaaagcaactgcgaagtatttcttttactcgaacatcatgaatttctttaagaccgagataacgctggcaaatggcgaaattagaaagaggcctctcatagagactaacggtgagacaggggaaatcgtctgggataagggtagggactttgcgacagtgcgcaaggtcctctctatgccgcaagttaatattgtgaagaaaaccgaggtgcagacgggaggcttctccaaggaaagcatacttcccaaacggaactctgataagttgatcgctcgtaagaaagattgggaccctaagaaatatggtgggttcgtttccccaactgttgcttacagcgtgctggtcgttgccaaggtcgagaagggtaaatccaagaaactcaaaagcgttaaggaactccttgggattactatcatggagagatcttcattcgaaaagaatcctatcgactttcttgaggccaaaggatataaggaagttaagaaagatctgataatcaaactcccaaagtactcattgtttgagctggaaaacggcaggaagcgcatgcttgcttccgccggagagttgcagaaagggaacgagttggctctgccttctaagtatgttaacttcctctatcttgcctctcattacgagaagctcaaaggctcaccagaggacaacgaacagaaacaactttttgtcgagcaacataagcactatttggatgagattatagaacagatcagtgaattctcgaaaagggttatccttgcagatgcgaatcttgacaaggtgttgtctgcatacaacaaacatagagataagccgatcagggagcaagcggaaaatatcattcacctcttcactcttacaaacttgggtgctcccgctgccttcaagtattttgataccacgattgaccggaaacaatacagatcaacgaaggaggtgctggatgccaccctcatccaccaatctattaccggactctacgagactagaatcgatctctcacagctcggcggggataaaagaccagcagcgacgaaaaaggcaggacaggctaagaagaagaaa

**c**

**VRERn(D10A) without TAG**

atggactacaaggaccacgacggggattacaaagaccacgacatagactacaaggatgacgatgacaaaatggcaccgaagaaaaaaaggaaggtcggaatccatggcgttccagctgccgataagaaatattccatcggactcgccattggcacgaatagcgtcggatgggctgttattactgatgagtacaaagttccgtctaagaagttcaaggtgctgggcaacacagaccgccacagcataaagaaaaatctcatcggtgcactccttttcgatagtggggagactgcagaagcgacaagattgaaaaggactgcgagaaggcgctatacacggcgtaagaatagaatctgctaccttcaggagattttctctaacgaaatggctaaggtcgatgacagtttctttcatagacttgaggaatcgttcttggttgaggaggataagaaacatgagaggcacccgatatttggaaacatcgtggatgaggtcgcatatcatgaaaagtaccccacaatctaccacctgagaaagaaactcgttgattccaccgacaaagcggatttgagactcatctacctcgctcttgcccatatgataaagttccgcggacactttctgatcgagggcgacctcaaccctgataatagcgacgtcgataagctcttcatccagttggttcaaacctacaatcagctctttgaggaaaacccaattaatgctagtggagtggatgcaaaagcgatactgtcggccagactctccaagagcagaaggttggagaacctgatcgctcaacttcctggagaaaagaaaaacggtctttttgggaatttgattgccttgtctctgggcctcacaccaaacttcaagtcaaattttgacctcgctgaggatgccaaacttcagttgtctaaggatacctatgatgacgatcttgacaatttgctggcacaaattggcgaccagtacgcggatctgttcctcgcagcgaagaatctgagtgatgctattctcctttcggacatactcagggttaacactgagatcacaaaagcacctttgagtgcgtcgatgattaagcgctatgatgaacatcaccaagacctcactttgctgaaggcccttgtgcggcagcaattgccagagaagtacaaagaaatcttctttgaccaatctaagaacggatacgctggctatattgatggaggagcttctcaggaggaattctataagtttatcaaacctatacttgagaagatggatggtacagaggaactccttgttaaattgaacagagaagatttgctgcgcaagcaacggacctttgacaacggatcaattccgcatcagatacacctcggcgagcttcatgccatccttcgccggcaggaagatttctacccctttttgaaggacaaccgcgagaagatagaaaaaatccttacgttccggattccttactatgtgggtccattggcaagggggaattcccgctttgcgtggatgactcggaaaagcgaggaaactatcacaccgtggaacttcgaggaagttgtggacaagggagcttctgcccaatcattcattgagaggatgactaacttcgataagaacctgccgaacgagaaagttctccccaagcactccctcctttacgagtatttcaccgtgtataacgaacttacgaaggttaaatacgtgactgagggtatgaggaagccagcattcttgagcggggaacaaaagaaagcgattgttgatttgctgtttaaaactaatcgcaaggtgacagtcaagcagctcaaagaggattatttcaagaaaattgaatgtttcgactctgtggagatatcaggagtcgaagataggtttaacgcttcccttggcacataccatgacctccttaagatcattaaggacaaagatttcctggataacgaggaaaatgaggacatcctcgaagatattgttcttaccttgacgctgtttgaggatcgcgaaatgatcgaggaacggcttaagacgtatgctcacttgttcgacgataaggttatgaagcagctcaagcgtagaaggtacactggatggggccgtctgtctagaaagctcatcaacggaatacgtgataaacaaagtggcaagacaattttggattttctgaagtcggacggattcgccaacagaaattttatgcagctgattcatgacgatagtctcaccttcaaagaggacatacagaaggctcaagtgagtggtcaaggggattcgctgcatgaacacatcgcaaacctcgcgggttcaccggccataaagaaaggaatccttcaaactgttaaggtcgttgatgagttggttaaagtgatgggtaggcacaagcccgaaaacatagtgatcgagatggctcgcgaaaatcagactacacaaaaagggcagaagaactctcgcgagcggatgaaaaggattgaggaaggaatcaaggaactgggctcacagattctcaaagagcatccagtcgaaaacacacagctgcaaaatgagaagctctatctttactatctccaaaatggccgggacatgtatgttgatcaggagcttgacatcaaccgtttgtccgactatgatgtggaccacattgtcccgcaatctttccttaaggacgattcaatcgataataaggtgttgacccggagcgataaaaaccgtggaaagtctgacaatgtcccttcagaggaagtggttaagaagatgaagaactactggagacaattgctgaatgcaaaactgatcacacagagaaagttcgacaacctcaccaaagcagagagaggtgggctcagtgaacttgataaagcgggcttcattaagcgtcagctcgttgagactagacagatcacgaagcatgtcgcgcagattttggattcgcggatgaacacgaagtacgacgagaatgataaactgatacgtgaagtcaaggttatcactcttaagtccaaattggtgagcgatttcagaaaggacttccaattctataaggtcagggagatcaacaattatcatcacgctcacgatgcctaccttaatgctgttgtggggaccgcccttattaagaaataccctaaattggagtctgaattcgtttacggggattataaggtctacgacgttaggaaaatgatagctaagagtgagcaggagatcggtaaagcaactgcgaagtatttcttttactcgaacatcatgaatttctttaagaccgagataacgctggcaaatggcgaaattagaaagaggcctctcatagagactaacggtgagacaggggaaatcgtctgggataagggtagggactttgcgacagtgcgcaaggtcctctctatgccgcaagttaatattgtgaagaaaaccgaggtgcagacgggaggcttctccaaggaaagcatacttcccaaacggaactctgataagttgatcgctcgtaagaaagattgggaccctaagaaatatggtgggttcgtttccccaactgttgcttacagcgtgctggtcgttgccaaggtcgagaagggtaaatccaagaaactcaaaagcgttaaggaactccttgggattactatcatggagagatcttcattcgaaaagaatcctatcgactttcttgaggccaaaggatataaggaagttaagaaagatctgataatcaaactcccaaagtactcattgtttgagctggaaaacggcaggaagcgcatgcttgcttccgcccgtgagttgcagaaagggaacgagttggctctgccttctaagtatgttaacttcctctatcttgcctctcattacgagaagctcaaaggctcaccagaggacaacgaacagaaacaactttttgtcgagcaacataagcactatttggatgagattatagaacagatcagtgaattctcgaaaagggttatccttgcagatgcgaatcttgacaaggtgttgtctgcatacaacaaacatagagataagccgatcagggagcaagcggaaaatatcattcacctcttcactcttacaaacttgggtgctcccgctgccttcaagtattttgataccacgattgaccggaaagagtacagatcaacgaaggaggtgctggatgccaccctcatccaccaatctattaccggactctacgagactagaatcgatctctcacagctcggcggggataaaagaccagcagcgacgaaaaaggcaggacaggctaagaagaagaaa

# Supplementary Figures and Table

## Supplementary Figures

**
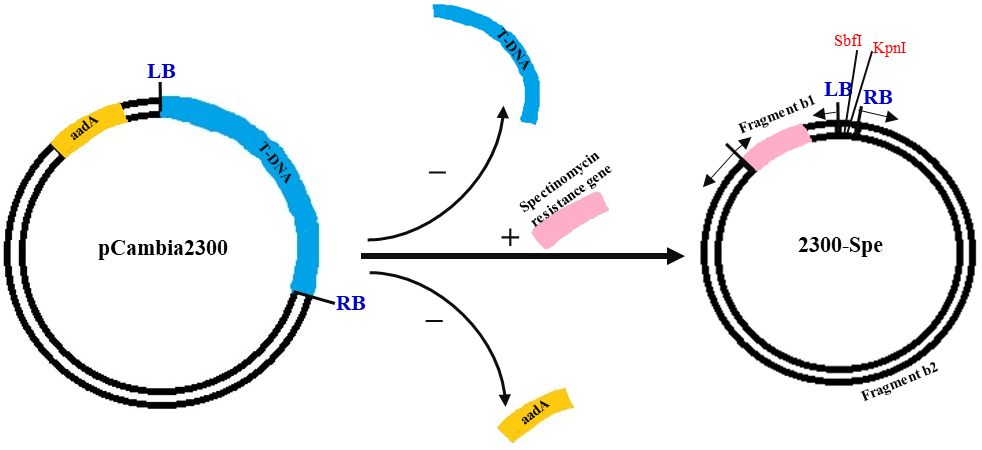
**

**Supplementary Figure S1 |** Schematic illustration of 2300-Spe vector construction. The whole T-DNA sequence in pCambia2300 was eliminated and the kanamycin resistance gene *aadA* was replaced with spectinomycin resistance gene in 2300-Spe. The 2300-Spe vector was generated by fusing Fragment b1 with Fragment b2 using an In-Fusion HD EcoDry Cloning Kit (Takara, Cat#639686) according to the manufacturer’s instructions. The fragments b1 and b2 were amplified by PCR with the primers listed in Supplementary Table S2. The spectinomycin resistance gene sequence was synthesized by GenScript Corp. (Nanjing, China). The restriction enzymes KpnI and SbfI were used to digest the 2300-Spe backbone. LB, left border. RB, right border.

**
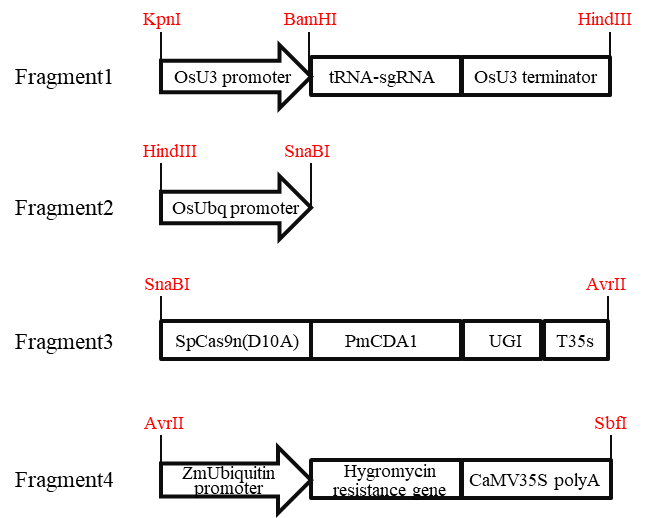
**

**Supplementary Figure S2 |** Schematic illustration of each fragment for construction of the PmCDA1-based cytosine base editor (pBEs), SpCas9n-pBE-basic vector. Fragment1 was synthesized by GenScript Corp. (Nanjing, China). For Fragment3, the *Streptococcus pyogenes* Cas9 nickase (SpCas9n (D10A)) sequence described in Cong et al. (2013) together with *Petromyzon marinus* cytidine deaminase 1 (PmCDA1) and uracil DNA glycosylase inhibitor (UGI) sequences were first codon-optimized for rice by GenScript Corp., then the whole sequence was synthesized with cauliflower mosaic virus 35S terminator (T35s). For Fragment2, the *Oryza sativa* ubiquitin promoter (OsUbq) was amplified from the *O. sativa* Nipponbare reference genome. The *Zea mays* ubiquitin promoter (ZmUbiquitin) was amplified from the B73 reference genome. The hygromycin resistance gene and the cauliflower mosaic virus 35S (CaMV35S) polyA were amplified from plasmid pCambia1300. Fragment4 was generated using an In-Fusion HD EcoDry Cloning Kit (Takara) according to the manufacturer’s instructions.

**
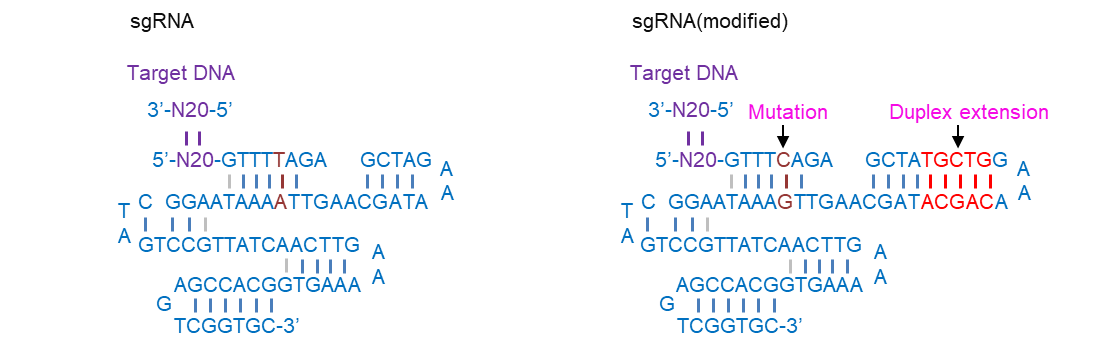
**

**Supplementary Figure S3 |** Nucleotide sequences of the single guide (sgRNA) and modified sgRNA. The mutation and introduced nucleotides are highlighted.

**Supplementary Figure S4 |** Comparison of indel frequencies in four target sites with NGAG PAMs induced by VQRn-pBE with the native sgRNA or modified sgRNA in rice T_0_ plants.

## Supplementary Tables

| **Supplementary Table S1 \|** Target sites and the corresponding vectors with the native sgRNA.   \| **target site** \| **Vector** \| **target site** \| **Vector** \| **target site** \| **Vector** \| **target site** \| **Vector** \| **target site** \| **Vector** \| \| --- \| --- \| --- \| --- \| --- \| --- \| --- \| --- \| --- \| --- \| \| W-T1 \| SpCas9n- pBE \| W-T7 \| VQRn-pBE-1 \| W-T8 \| VQRn-pBE-2 \| W-T11 \| VQRn-pBE-3 \| W-T13 \| VQRn-pBE-4 \| \| W-T2 \| W-T9 \| W-T10 \| W-T12 \| W-T14 \| \| W-T3 \| ALS-T1 \| ALS-T4 \| W-T19 \| W-T15 \| \| W-T4 \| ALS-T2 \| ALS-T5 \| W-T20 \| W-T16 \| \| W-T5 \| ALS-T3 \| ALS-T6 \| W-T21 \| W-T17 \| \| W-T6 \|  \|  \| W-T22 \| W-T18 \|   **Supplementary Table S2 \|** Primers used in this study.   \| **Primer name** \| **Primer sequence (5’-3’)** \| **Purpse** \| \| --- \| --- \| --- \| \| Fragment b1-F \| ggtaccagctcaagctgctcctgcaggtcgactccgattcgg \| Amplifying Fragment b1 \| \| Fragment b1-R \| catgcattctaggtattatttgccgactactttggtgatc \| \| Fragment b2-F \| tacctagaatgcatgaccaaaatcccttaacgtgagt \| Amplifying Fragment b2 \| \| Fragment b2-R \| gcttgagctggtacctggatcagattgtcgtttccc \| \| prOsUbq-F \| tgctaagcttacaaattcgggtcaaggcgg \| Amplifying OsUbq promoter and Fragment 2 \| \| prOsUbq-R \| ccattacgtactgcaagaaataatcaccaa \| \| prZmUbi1-F \| tccgcctaggctgcagtgcagcgtgacc \| Amplifying ZmUbiquitin promoter; prZmUbi1-F used for Fragment 4 \| \| prZmUbi1-R \| tcatgagctcctgcagaagtaacaccaaacaac \| \| Hpt-F \| gcaggagctcatgaaaaagcctgaactcaccgcg \| Amplifying Hygromycin resistance gene and CaMV35S polyA; T35S-R1 used for Fragment 4 \| \| T35S-R1 \| gcgtcctgcaggctgaattaacgccgaattaa \| \| prOsUbq-3’ \| attatgtagcttgtgcgtttcg \| Transgenic calli and T_0_ plants detection \| \| SpCas9-5’ \| gatgaagagcttatcgacgt \| \| NAG-F1 \| agtcagggtctcttgcatgacactggagttgattacagttttagagctagaaatagcaa \| Construction of SpCas9n -pBE with targets \| \| NAG-F2 \| atcgatggtctcacacggtcgactccacgcttggttttagagctagaaatagcaa \| \| NAG-F3 \| atcgatggtctcactcctcggtacgaccagtacgttttagagctagaaatagcaa \| \| NAG-F4 \| atcgatggtctcaagccacaacgctggtatcccgttttagagctagaaatagcaa \| \| NAG-F5 \| atcgatggtctcatggcacactggcccactggcgttttagagctagaaatagcaa \| \| NAG-R1 \| atcgatggtctcccgtgtgcaccagccgggaatcgaac \| \| NAG-R2 \| atcgatggtctcaggagtgcaccagccgggaatcgaac \| \| NAG-R3 \| atcgatggtctctggcttgcaccagccgggaatcgaac \| \| NAG-R4 \| atcgatggtctctgccatgcaccagccgggaatcgaac \| \| NAG-R5 \| agtcatggtctcgaaaaccaacgactggcacactggcctgcaccagccgggaatcgaac \| \| NAG-F6 \| agtcagggtctcttgcatgacactggagttgattaca gtttcagagctatgctggaaac \| \| NAG-F7 \| atcgatggtctcacacggtcgactccacgcttg gtttcagagctatgctggaaac \| \| NAG-F8 \| atcgatggtctcactcctcggtacgaccagtac gtttcagagctatgctggaaac \| \| NAG-F9 \| atcgatggtctcaagccacaacgctggtatccc gtttcagagctatgctggaaac \| \| NAG-F10 \| atcgatggtctcatggcacactggcccactggcgtttcagagctatgctggaaac \| \| NAG-R6 \| atcgatggtctcccgtgtgcaccagccgggaatcgaac \| \| NAG-R7 \| atcgatggtctcaggagtgcaccagccgggaatcgaac \| \| NAG-R8 \| atcgatggtctctggcttgcaccagccgggaatcgaac \| \| NAG-R9 \| atcgatggtctctgccatgcaccagccgggaatcgaac \| \| NAG-R10 \| agtcatggtctcagaaaccaacgactggcacactggcctgcaccagccgggaatcgaac \| \| VQRn-F1 \| ggtgggttcgtttccccaactgttgcttacagcgtgctgg \| VQRn mutation \| \| VQRn-R1 \| gatctgtattgtttccggtcaatcgtggtatcaaaatacttg \| \| VQRn-F2 \| gaccggaaacaatacagatcaacgaaggaggtgctggatg \| \| VQRn-R2 \| gttggggaaacgaacccaccatatttcttagggtcccaatctttc \| \| VRERn-F1 \| ggtgggttcgtttccccaactgttgcttacagcgtgctgg \| VRERn mutation \| \| VRERn-R1 \| ctgcaactcacgggcggaagcaagcatgcgcttcctgc \| \| VRERn-F2 \| cttccgcccgtgagttgcagaaagggaacgagttggctct \| \| VRERn-R2 \| gatctgtactctttccggtcaatcgtggtatcaaaatacttg \| \| VRERn-F3 \| gaccggaaagagtacagatcaacgaaggaggtgctggatg \| \| VRERn-R3 \| gttggggaaacgaacccaccatatttcttagggtcccaatc \| \| VRER-F1 \| Actacaggtctcctgcagccacaacgctggtatcccagttttagagctagaaatagcaa \| Construction of VRERn-pBE with targets \| \| VRER-R1 \| actacaggtctccatcatgcaccagccgggaa \| \| VRER-F2 \| actacaggtctcctgattacaaagacaaccagagttttagagctagaaatagcaa \| \| VRER-R2 \| actacaggtctccagtctgcaccagccgggaa \| \| VRER-F3 \| actacaggtctccgactggcacactggcccactgttttagagctagaaatagcaa \| \| VRER-R3 \| actacaggtctccgagatgcaccagccgggaa \| \| VRER-F4 \| actacaggtctcctctccggcatcgccaggggagttttagagctagaaatagcaa \| \| VRER-R4 \| actacaggtctccaaaacgacgtccatgccgttgacgatgcaccagccgggaa \| \| VRER-F5 \| actacaggtctcctgcaccggtcaccccgccgtccccgttttagagctagaaatagcaa \| \| VRER-R5 \| actacaggtctccacgctgcaccagccgggaa \| \| VRER-F6 \| actacaggtctccgcgtccatggagatccaccagttttagagctagaaatagcaa \| \| VRER-R6 \| actacaggtctccaggttgcaccagccgggaa \| \| VRER-F7 \| actacaggtctccacctcttccgccacgagcaggttttagagctagaaatagcaa \| \| VRER-R7 \| actacaggtctccagcctgcaccagccgggaa \| \| VRER-F8 \| actacaggtctccggctgtcttcggctggtctggttttagagctagaaatagcaa \| \| VRER-R8 \| actacaggtctcccccatgcaccagccgggaa \| \| VRER-F9 \| actacaggtctcctgggcaacccggaatgtgaggttttagagctagaaatagcaa \| \| VRER-R9 \| actacaggtctccaaaaccccacttgggatcataggcatgcaccagccgggaa \| \| VQR-F1 \| actacaggtctcctgcatgcagacaggtacgagaggggttttagagctagaaatagcaa \| Construction of VQRn-pBE with targets \| \| VQR-R1 \| actacaggtctccctggtgcaccagccgggaa \| \| VQR-F2 \| actacaggtctccccagcgttgtggctgaggtagttttagagctagaaatagcaa \| \| VQR-R2 \| actacaggtctcccgattgcaccagccgggaa \| \| VQR-F3 \| actacaggtctccatcgaccatccgtcattcctgttttagagctagaaatagcaa \| \| VQR-R3 \| actacaggtctccagcttgcaccagccgggaa \| \| VQR-F4 \| actacaggtctccagcttcctcatgaacattcagttttagagctagaaatagcaa \| \| VQR-R4 \| actacaggtctcctgcctgcaccagccgggaa \| \| VQR-F5 \| actacaggtctccggcaacccggaatgtgagaggttttagagctagaaatagcaa \| \| VQR-R5 \| actacaggtctccaaaactctttgttacacggactgcatgcaccagccgggaa \| \| VQR-F6 \| actacaggtctcctgcatttccattgctacaagcgtgttttagagctagaaatagcaa \| \| VQR-R6 \| actacaggtctccacggtgcaccagccgggaatcg \| \| VQR-F7 \| actacaggtctccccgtcattcctggagaaggtgttttagagctagaaatagcaa \| \| VQR-R7 \| actacaggtctccggagtgcaccagccgggaa \| \| VQR-F8 \| actacaggtctccctccggccgtgggggccggcgttttagagctagaaatagcaa \| \| VQR-R8 \| actacaggtctccggtatgcaccagccgggaa \| \| VQR-F9 \| actacaggtctcctacccgggcggcgcgtccatgttttagagctagaaatagcaa \| \| VQR-R9 \| actacaggtctccaaaactggcggaagaggtggttggttgcaccagccgggaa \| \| VQR-F10 \| agtcagggtctcttgcaccagcgttgtggctgaggtagtttcagagctatgctggaaac \| \| VQR-F11 \| Atcgatggtctcatgcagacaggtacgagaggggtttcagagctatgctggaaac \| \| VQR-F12 \| atcgatggtctcatttccattgctacaagcgtgtttcagagctatgctggaaac \| \| VQR-F13 \| atcgatggtctcaatcgaccatccgtcattcctgtttcagagctatgctggaaac \| \| VQR-R10 \| atcgatggtctcctgcatgcaccagccgggaatcgaac \| \| VQR-R11 \| atcgatggtctcggaaatgcaccagccgggaatcgaac \| \| VQR-R12 \| atcgatggtctctcgattgcaccagccgggaatcgaac \| \| VQR-R13 \| agtcatggtctcagaaacaccttctccaggaatgacggtgcaccagccgggaatcgaac \| \| VQR-F14 \| agtcagggtctcttgcaacaacaacccatacttcaaagttttagagctagaaatagcaa \| \| VQR-F15 \| atcgatggtctcatctgcaaccttgatctgcaagttttagagctagaaatagcaa \| \| VQR-F16 \| atcgatggtctcagactccacgcttgtagcaatgttttagagctagaaatagcaa \| \| VQR-F17 \| atcgatggtctcacaccggtctttccccaaaccgttttagagctagaaatagcaa \| \| VQR-F18 \| atcgatggtctcatacggacctgacactggagtgttttagagctagaaatagcaa \| \| VQR-R14 \| atcgatggtctcgcagatgcaccagccgggaatcgaac \| \| VQR-R15 \| atcgatggtctcgagtctgcaccagccgggaatcgaac \| \| VQR-R16 \| atcgatggtctccggtgtgcaccagccgggaatcgaac \| \| VQR-R17 \| atcgatggtctcccgtatgcaccagccgggaatcgaac \| \| VQR-R18 \| agtcatggtctcgaaaacaggttgcagacaggtacgagtgcaccagccgggaatcgaac \| \| VQR-F19 \| agtcagggtctcttgcactccaggaatgacggatggtgttttagagctagaaatagcaa \| \| VQR-F20 \| atcgatggtctcaaactccagtgtcaggtccgtgttttagagctagaaatagcaa \| \| VQR-F21 \| atcgatggtctcacattgctacaagcgtggagtgttttagagctagaaatagcaa \| \| VQR-F22 \| atcgatggtctcaagaccggtgagaagatctacgttttagagctagaaatagcaa \| \| VQR-F23 \| atcgatggtctcagacactggagttgattacaagttttagagctagaaatagcaa \| \| VQR-R19 \| atcgatggtctcgagtttgcaccagccgggaatcgaac \| \| VQR-R20 \| atcgatggtctccaatgtgcaccagccgggaatcgaac \| \| VQR-R21 \| atcgatggtctcggtcttgcaccagccgggaatcgaac \| \| VQR-R22 \| atcgatggtctcgtgtctgcaccagccgggaatcgaac \| \| VQR-R23 \| agtcatggtctcgaaaactggtgatctctcctcggtactgcaccagccgggaatcgaac \| \| VQR-F24 \| agtcagggtctcttgcaacaacaacccatacttcaaagtttcagagctatgctggaaac \| \| VQR-F25 \| atcgatggtctcatctgcaaccttgatctgcaagtttcagagctatgctggaaac \| \| VQR-F26 \| atcgatggtctcagactccacgcttgtagcaatgtttcagagctatgctggaaac \| \| VQR-F27 \| atcgatggtctcacaccggtctttccccaaaccgtttcagagctatgctggaaac \| \| VQR-F28 \| Atcgatggtctcatacggacctgacactggagtgtttcagagctatgctggaaac \| \| VQR-R24 \| atcgatggtctcgcagatgcaccagccgggaatcgaac \| \| VQR-R25 \| atcgatggtctcgagtctgcaccagccgggaatcgaac \| \| VQR-R26 \| atcgatggtctccggtgtgcaccagccgggaatcgaac \| \| VQR-R27 \| atcgatggtctcccgtatgcaccagccgggaatcgaac \| \| VQR-R28 \| agtcatggtctcagaaacaggttgcagacaggtacgagtgcaccagccgggaatcgaac \| \| VQR-F29 \| agtcagggtctcttgcactccaggaatgacggatggtgtttcagagctatgctggaaac \| \| VQR-F30 \| atcgatggtctcaaactccagtgtcaggtccgtgtttcagagctatgctggaaac \| \| VQR-F31 \| atcgatggtctcacattgctacaagcgtggagtgtttcagagctatgctggaaac \| \| VQR-F32 \| atcgatggtctcaagaccggtgagaagatctacgtttcagagctatgctggaaac \| \| VQR-F33 \| atcgatggtctcagacactggagttgattacaagtttcagagctatgctggaaac \| \| VQR-R29 \| atcgatggtctcgagtttgcaccagccgggaatcgaac \| \| VQR-R30 \| atcgatggtctccaatgtgcaccagccgggaatcgaac \| \| VQR-R31 \| atcgatggtctcggtcttgcaccagccgggaatcgaac \| \| VQR-R32 \| atcgatggtctcgtgtctgcaccagccgggaatcgaac \| \| VQR-R33 \| agtcatggtctcagaaactggtgatctctcctcggtactgcaccagccgggaatcgaac \| \| T7-OT1-F \| gcataaaccccacaaaatgg \| Detection of off-target mutations \| \| T7-OT1-R \| tccgataaaccctcatcgtc \| \| T7-OT2-F \| cattgatggtgcaggagaca \| \| T7-OT2-R \| tgcagagcatctggatttga \| \| T7-OT3-F \| caaatcacccacgtcaacag \| \| T7-OT3-R \| aggtagagccctgcttccac \| \| T8-OT1-F \| ggcattggcgtacaaaactt \| \| T8-OT1-R \| tggccttctccttgatcttg \| \| T8-OT2-F \| gcgcatgatgtgaaacaatc \| \| T8-OT2-R \| gccaaatgggtgtcaagaat \| \| T8-OT3-F \| tgtgctcaccgagaactttg \| \| T8-OT3-R \| tgcattcatgtagtcgcagag \| \| T9-OT1-F \| cgaaccttggtgctagggta \| \| T9-OT1-R \| tcccacaataccgatcttcc \| \| T9-OT2-F \| ttttcccattggatttgctg \| \| T9-OT2-R \| tccacattagctccatgcac \| \| T9-OT3-F \| ccatctctctccctgcaaag \| \| T9-OT3-R \| agctcccaagagatgcattg \| \| T9-OT4-F \| actccggcacctcttctaca \| \| T9-OT4-R \| caagtccccaagtccagttc \|   **Supplementary Table S3 \|** Targets used to determine the effects of SpCas9n-pBE in resistant rice calli. | | | | |
| --- | --- | --- | --- | --- | --- | --- | --- | --- | --- | --- | --- | --- | --- | --- | --- | --- | --- | --- | --- | --- | --- | --- | --- | --- | --- | --- | --- | --- | --- | --- | --- | --- | --- | --- | --- | --- | --- | --- | --- | --- | --- | --- | --- | --- | --- | --- | --- | --- | --- | --- | --- | --- | --- | --- | --- | --- | --- | --- | --- | --- | --- | --- | --- | --- | --- | --- | --- | --- | --- | --- | --- | --- | --- | --- | --- | --- | --- | --- | --- | --- | --- | --- | --- | --- | --- | --- | --- | --- | --- | --- | --- | --- | --- | --- | --- | --- | --- | --- | --- | --- | --- | --- | --- | --- | --- | --- | --- | --- | --- | --- | --- | --- | --- | --- | --- | --- | --- | --- | --- | --- | --- | --- | --- | --- | --- | --- | --- | --- | --- | --- | --- | --- | --- | --- | --- | --- | --- | --- | --- | --- | --- | --- | --- | --- | --- | --- | --- | --- | --- | --- | --- | --- | --- | --- | --- | --- | --- | --- | --- | --- | --- | --- | --- | --- | --- | --- | --- | --- | --- | --- | --- | --- | --- | --- | --- | --- | --- | --- | --- | --- | --- | --- | --- | --- | --- | --- | --- | --- | --- | --- | --- | --- | --- | --- | --- | --- | --- | --- | --- | --- | --- | --- | --- | --- | --- | --- | --- | --- | --- | --- | --- | --- | --- | --- | --- | --- | --- | --- | --- | --- | --- | --- | --- | --- | --- | --- | --- | --- | --- | --- | --- | --- | --- | --- | --- | --- | --- | --- | --- | --- | --- | --- | --- | --- | --- | --- | --- | --- | --- | --- | --- | --- | --- | --- | --- | --- | --- | --- | --- | --- | --- | --- | --- | --- | --- | --- | --- | --- | --- | --- | --- | --- | --- | --- | --- | --- | --- | --- | --- | --- | --- | --- | --- | --- | --- | --- | --- | --- | --- | --- | --- | --- | --- | --- | --- | --- | --- | --- | --- | --- | --- | --- | --- | --- | --- | --- | --- | --- | --- | --- | --- | --- | --- | --- | --- | --- | --- | --- | --- | --- | --- | --- | --- | --- | --- | --- | --- | --- | --- | --- | --- | --- | --- | --- | --- | --- | --- | --- | --- | --- | --- | --- | --- | --- | --- | --- | --- | --- | --- | --- | --- | --- | --- | --- | --- | --- |
| **target site** | **gene** | **sequence** | **PAM** | **Indel frequency**  **in calli (%)** |
| W-T1 | *OsWaxy* | ctcctcggtacgaccagtac | NAG | 0 |
| W-T2 | *OsWaxy* | agccacaacgctggtatccc | NAG | 0 |
| W-T3 | *OsWaxy* | tggcacactggcccactggc | NAG | 0 |
| W-T4 | *OsWaxy* | tgacactggagttgattaca | NAG | 0 |
| W-T5 | *OsWaxy* | cacggtcgactccacgcttg | NAG | 0 |
| W-T6 | *OsWaxy* | ggccagtgtgccagtcgttg | NAG | 0 |

| **Supplementary Table S4 \|** Frequencies of indels in the *OsWaxy* gene induced by SpCas9n-pBE in T_0_ plants. | | | |
| --- | --- | --- | --- |
| Target site | PAM | Indel frequency (%) | T_0_ plant No. of different C-to-T genotypes |
| W-T1 | NAG | 20.0 | C1>T1(1);C3>T3(1);C3C4>T3T4(2);C1C3C4C6>T1T3T4T6(1) |
| W-T2 |  | 0 | C3C4>T3T4(1) |
| W-T3 |  | 0 | C13>T13(1) |
| W-T4 |  | 0 |  |
| W-T5 |  | 0 |  |
| W-T6 |  | 0 | C3>T3(2);C3C4>T3T4(4);C3C4C12>T3T4T12(1) |

**Supplementary Table S5 |** Mutations induced by SpCas9n-pBE at six target sites in each T_0_ transgenic line.

| T_0_ plants | W-T1 | W-T2 | W-T3 | W-T4 | W-T5 | W-T6 |  |  |
| --- | --- | --- | --- | --- | --- | --- | --- | --- |
| Line1 | M | W | Null | W | W | Null |  |  |
| Line2 | W | W | W | W | W | W |  |  |
| Line3 | W | W | M | W | W | M |  |  |
| Line4 | W | W | Null | W | W | M |  |  |
| Line5 | M | W | Null | W | W | Null |  |  |
| Line6 | W | Null | Null | Null | Null | W |  |  |
| Line7 | W | W | W | W | W | M |  | M: Base mutation |
| Line8 | M | Null | Null | Null | Null | M |  | W: Wild type |
| Line9 | W | W | W | W | W | W |  | Null: Failed to detect |
| Line10 | W | W | W | W | W | W |  |  |
| Line11 | M | M | W | W | W | M |  |  |
| Line12 | W | W | W | W | W | M |  |  |
| Line13 | W | W | W | W | W | W |  |  |
| Line14 | W | W | W | W | W | W |  |  |
| Line15 | M | W | W | W | W | M |  |  |

| **Supplementary Table S6 \|** Target sites used to determine the effects of VRERn-pBE in resistant rice calli. | | | | |
| --- | --- | --- | --- | --- |
| **target site** | **gene** | **sequence** | **PAM** | **C-to-T frequency in calli (%)** |
| 1 | *OsWaxy* | gccacaacgctggtatccca | NGCG | 0 |
| 2 | *OsWaxy* | tgattacaaagacaaccaga | NGCG | 0 |
| 3 | *OsWaxy* | gactggcacactggcccact | NGCG | 0 |
| 4 | *OsWaxy* | tctccggcatcgccagggga | NGCG | 0 |
| 5 | *OsWaxy* | tcgtcaacggcatggacgtc | NGCG | 0 |
| 6 | *OsALS* | ccggtcaccccgccgtcccc | NGCG | 0 |
| 7 | *OsALS* | gcgtccatggagatccacca | NGCG | 0 |
| 8 | *OsALS* | acctcttccgccacgagcag | NGCG | 0 |
| 9 | *OsALS* | ggctgtcttcggctggtctg | NGCG | 0 |
| 10 | *OsALS* | tgggcaacccggaatgtgag | NGCG | 0 |
| 11 | *OsALS* | tgcctatgatcccaagtggg | NGCG | 0 |

| **Supplementary Table S7** \| Target sites used to determine the effects of VQRn-pBE. | | | |
| --- | --- | --- | --- |
| **target site** | **gene** | **sequence** | **PAM** |
| W-T7 | *OsWaxy* | tgcagacaggtacgagaggg | NGAG |
| W-T8 | *OsWaxy* | ccgtcattcctggagaaggt | NGAG |
| W-T9 | *OsWaxy* | ccagcgttgtggctgaggta | NGAG |
| W-T10 | *OsWaxy* | tttccattgctacaagcgt | NGAG |

**Supplementary Table S8 |** Potential off-target sites analyzed for three endogenous genomic loci.

| **Targets** | **Chromosome** | **Position** | **Sequence (5’-3’)** | **Mismatches number** |
| --- | --- | --- | --- | --- |
| W-T7 | chr06 | 1767569 | tgcagacaggtacgagaggg | 0 |
| W-T7-OT1 | chr03 | 20839752 | tgcGgaAGggtacgagaggg | 3 |
| W-T7-OT2 | chr05 | 21454212 | tgATgacaTgtacgagagAg | 4 |
| W-T7-OT3 | chr04 | 33730023 | AgcagacaggAaGgagagAg | 4 |
| W-T8 | chr06 | 1767642 | ccgtcattcctggagaaggt | 0 |
| W-T8-OT1 | chr05 | 21131220 | ccTtcattccATgagaaggt | 3 |
| W-T8-OT2 | chr12 | 18471089 | ccgtGattGctgAagaaAgt | 4 |
| W-T8-OT3 | chr05 | 20900865 | GTgtcatCActgCagaaggt | 5 |
| W-T9 | chr06 | 1767437 | ccagcgttgtggctgaggta | 0 |
| W-T9-OT1 | chr08 | 15507385 | ccagcgtGgtggGCgaggCa | 4 |
| W-T9-OT2 | chr01 | 16386221 | ccaTcgttgtgTctgaggAa | 3 |
| W-T9-OT3 | chr03 | 28747147 | ccagTCttgtggTtTaggta | 4 |
| W-T9-OT4 | chr04 | 22377510 | ccGgcgttgtCgcACaggta | 4 |

| **Supplementary Table S9 \|** Target sites in the *OsALS* gene selected to determine the effects of VQRn-pBE. | | |
| --- | --- | --- |
| **target site** | **gene** | **sequence** |
| ALS-T1 | *OsALS* | agcttcctcatgaacattca |
| ALS-T2 | *OsALS* | ggcaacccggaatgtgagag |
| ALS-T3 | *OsALS* | tgcagtccgtgtaacaaaga |
| ALS-T4 | *OsALS* | ctccggccgtgggggccggc |
| ALS-T5 | *OsALS* | tacccgggcggcgcgtccat |
| ALS-T6 | *OsALS* | accaaccacctcttccgcca |

| **Supplementary Table S10 \|** Target sites in the *OsWaxy* gene used to determine the effects of VQRn-pBE. | | | |
| --- | --- | --- | --- |
| **target site** | **gene** | **sequence** | **PAM** |
| W-T11 | *OsWaxy* | tacggacctgacactggagt | NGAT |
| W-T12 | *OsWaxy* | ctcgtacctgtctgcaacct | NGAT |
| W-T13 | *OsWaxy* | ctccaggaatgacggatggt | NGAT |
| W-T14 | *OsWaxy* | aactccagtgtcaggtccgt | NGAT |
| W-T15 | *OsWaxy* | gtaccgaggagagatcacca | NGAC |
| W-T16 | *OsWaxy* | cattgctacaagcgtggagt | NGAC |
| W-T17 | *OsWaxy* | agaccggtgagaagatctac | NGAC |
| W-T18 | *OsWaxy* | gacactggagttgattacaa | NGAC |
| W-T19 | *OsWaxy* | acaacaacccatacttcaaa | NGAA |
| W-T20 | *OsWaxy* | tctgcaaccttgatctgcaa | NGAA |
| W-T21 | *OsWaxy* | gactccacgcttgtagcaat | NGAA |
| W-T22 | *OsWaxy* | caccggtctttccccaaacc | NGAA |

| **Supplementary Table S11 \|** Frequencies of mutations in the *OsWaxy* gene induced by VQRn-pBE in T_0_ plants. | | | | |
| --- | --- | --- | --- | --- |
| Target site | PAM | Indel frequency  (%) | T_0_ plant No. of different C-to-T genotypes | No. of Heterozygous/ Homozygous |
| W-T11 | VQR-NGAT | 0 | C3>T3(1) | 1/0 |
| W-T12 |  | 0 | C3>T3(1) | 1/0 |
| W-T13 |  | 0 | C1C3C4>T1T3T4(1) | 1/0 |
| W-T14 |  | 0 |  |  |
| W-T15 | VQR-NGAC | 14.3 | C5>T5(2);C4C5>T4T5(5) | 6/1 |
| W-T16 |  | 0 |  |  |
| W-T17 |  | 0 |  |  |
| W-T18 |  | 0 |  |  |
